# Enhancing Lentiviral and Alpharetroviral Transduction of Human Hematopoietic Stem Cells for Clinical Application

**DOI:** 10.1016/j.omtm.2019.05.015

**Published:** 2019-06-07

**Authors:** Juliane W. Schott, Diego León-Rico, Carolina B. Ferreira, Karen F. Buckland, Giorgia Santilli, Myriam A. Armant, Axel Schambach, Alessia Cavazza, Adrian J. Thrasher

**Affiliations:** 1 Infection, Immunity and Inflammation Program, Molecular and Cellular Immunology Section, UCL Great Ormond Street Institute of Child Health, University College London, London WC1N 1EH, UK; 2 Division of Hematology/Oncology, Boston Children’s Hospital, Harvard Medical School, Boston, MA 02115, USA; 3 Institute of Experimental Hematology, Hannover Medical School, 30625 Hannover, Germany; 4 Great Ormond Street Hospital NHS Foundation Trust, London WC1N 1EH, UK

**Keywords:** lentiviral vector, alpharetroviral vector, CD34^+^ cell transduction, HSPC, transduction enhancer, gene therapy, ATMP, SCID-X1, LentiBOOST, protamine sulfate

## Abstract

*Ex vivo* retroviral gene transfer into CD34^+^ hematopoietic stem and progenitor cells (HSPCs) has demonstrated remarkable clinical success in gene therapy for monogenic hematopoietic disorders. However, little attention has been paid to enhancement of culture and transduction conditions to achieve reliable effects across patient and disease contexts and to maximize potential vector usage and reduce treatment cost. We systematically tested three HSPC culture media manufactured to cGMP and eight previously described transduction enhancers (TEs) to develop a state-of-the-art clinically applicable protocol. Six TEs enhanced lentiviral (LV) and five TEs facilitated alpharetroviral (ARV) CD34^+^ HSPC transduction when used alone. Combinatorial TE application tested with LV vectors yielded more potent effects, with up to a 5.6-fold increase in total expression of a reporter gene and up to a 3.8-fold increase in VCN. Application of one of the most promising combinations, the poloxamer LentiBOOST and protamine sulfate, for GMP-compliant manufacturing of a clinical-grade advanced therapy medicinal product (ATMP) increased total VCN by over 6-fold, with no major changes in global gene expression profiles or inadvertent loss of CD34^+^CD90^+^ HSPC populations. Application of these defined culture and transduction conditions is likely to significantly improve *ex vivo* gene therapy manufacturing protocols for HSPCs and downstream clinical efficacy.

## Introduction

Retroviral-vector-mediated gene therapy has demonstrated remarkable clinical success over the past 2 decades.^1^, ^2^, ^3^, ^4^, ^5^, ^6^, ^7^, ^8^, ^9^, ^10^, ^11^, ^12^, ^13^ Disorders of the hematopoietic system are especially suitable for gene therapy due to the unique hierarchy of the blood system, with the hematopoietic stem cell (HSC) being able to reconstitute all blood lineages and self-renew, providing potential for a lifelong cure. Also, HSCs are readily accessible from bone marrow or from peripheral blood upon pharmacological mobilization. The *ex vivo* modification of hematopoietic stem and progenitor cells (HSPCs) requires *in vitro* culture of isolated HSPCs over a period of several days. Applied culture conditions can significantly affect HSC maintenance, expansion, and quality, which, in turn, determine engraftment capacity, differentiation potential, and long-term stem cell performance upon reinfusion into the patient. Currently, a number of different commercially available culture media manufactured to current good manufacturing practice (cGMP) are being differentially used across treatment centers, with little standardization.

While early trials using long terminal repeat (LTR)-driven gammaretroviral vectors were overshadowed by the occurrence of insertional mutagenesis and leukemia caused by the integrated vector,^14^, ^15^, ^16^, ^17^, ^18^ introduction of the self-inactivating (SIN) design to remove strong enhancer and promoter elements from the vector’s LTRs,^19^, ^20^ and/or switch to the application of lentiviral (LV) vectors,^21^ which display a more favorable integration pattern,^22^ has so far demonstrated safety in clinical trials.^3^, ^4^, ^5^, ^7^, ^9^, ^10^, ^12^, ^13^ Alpharetroviral (ARV) vectors are an emerging tool for gene therapy and have entered preclinical testing.^23^, ^24^, ^25^ Inherent features of this vector class render them potentially superior to other retroviral vector family members in terms of safety and, thus, promising for future clinical application. These features are (1) a short leader region devoid of any major splice sites and any overlap with retroviral coding regions^23^ and (2) the most neutral integration pattern among the different retroviral vectors developed so far.^24^, ^25^, ^26^

Despite the success of retrovirus-based CD34^+^ HSPC gene therapy for several hematopoietic and non-hematopoietic indications, HSPC transduction remains challenging and cannot be achieved across all patients and disease contexts. Some disorders, in particular, require high copy numbers or a high proportion of gene-modified cells. As CD34^+^ HSPCs are known to be relatively difficult to transduce, high MOIs are applied to overcome existing barriers and achieve clinically relevant transduction levels. For these reasons, clinical protocols would benefit from the identification of conditions for enhanced transduction, allowing greater predictability of dosing and reduced cost of goods due to more efficient use of vector lots. A continuously expanding list of small molecule compounds and peptides acting as transduction enhancers (TEs) have been identified. Mechanistically, these can be grouped into two major categories: (1) entry enhancers, which physically increase co-localization of or lower the repulsion between viral vector particles and target cells, or which trigger fusion (RetroNectin,^27^, ^28^ LentiBOOST,^29^ protamine sulfate (PS),^30^ Vectofusin-1,^31^, ^32^ ViraDuctin, and staurosporine [Stauro]^33^), and (2) post-entry TEs, ultimately yielding higher copy numbers of integrated vector by affecting intracellular processes, such as prostaglandin E2 (PGE2).^34^ While the beneficial effect of each individual compound has been previously demonstrated,^27^, ^28^, ^29^, ^30^, ^31^, ^32^, ^33^, ^34^ a side-by-side comparison to identify TEs or combinations of TEs with the greatest potential for CD34^+^ HSPC transduction remains elusive.

To improve current clinical protocols for *ex vivo* HSPC clinical gene therapy, we systematically compared different HSPC culture media manufactured to cGMP as well as the effects of previously described TEs on both LV and ARV transduction efficiency. The conditions identified as most suitable were subsequently applied to a GMP-compliant manufacturing process of an HSPC advanced therapy medicinal product (ATMP) for treatment of X-linked severe combined immunodeficiency (SCID-X1).

## Results

### Media Comparison for CD34^+^ HSPC Culture

**Figure 1:**
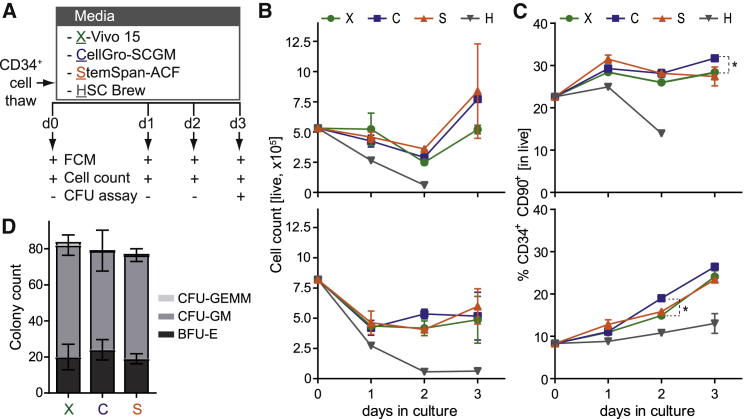
Media Comparison for CD34^+^ HSPC Culture and Expansion (A) Experimental scheme. Purified CD34^+^ HSPCs from 2 different HDs were thawed and cultured in X-Vivo 15 (green), SCGM (blue), StemSpan (orange) and HSC Brew (gray) in the presence of SCF (300 ng/mL), Flt3-L (300 ng/mL), and TPO (100 ng/mL). Cultures were analyzed daily by FCM for cell counts and expression of stem cell markers. Cells were additionally subjected to colony-forming unit (CFU) assay on day 3 post-thaw. (B and C) Cell counts assessed with counting beads (B) and percentage of CD34^+^CD90^+^ HSPCs^prim^ (C), determined by FCM at the indicated time points of culture in the different media. Top: donor A; bottom: donor B. Error bars indicate the mean of 2 independent technical duplicates ± SD; *adjusted p ≤ 0.05, two-way ANOVA with Geisser-Greenhouse correction. (D) Number of BFU-E, CFU-GM, and CFU-GEMM colonies in the CFU assay, determined after 2 weeks of culture in MethoCult. HSC Brew was excluded from CFU assay due to insufficient cell numbers.

To define optimal HSPC culture conditions, we compared three different commercially available culture media manufactured to cGMP (X-Vivo 15, stem cell growth medium [SCGM], and HSC Brew). For reference, we included the animal-component-free (ACF) version of StemSpan, used extensively in preclinical research. Purified CD34^+^ HSPCs from two healthy donors (HDs) were cultured in the different media according to a standard clinical protocol (Figure 1A). Cultures were monitored daily by flow cytometry (FCM) determining cell viability, cell counts, and the HSPC percentage. A common marker profile beyond CD34 to phenotypically discriminate more primitive HSPCs is CD34^+^CD90^+^CD38^−^.^35^ However, due to progressive *in vitro* decrease of CD38 expression as a culture artifact,^36^, ^37^ we omitted this marker from our analyses, defining HSPCs^prim^ by co-expression of the CD34 and the CD90 surface antigens (Figure S1A).

Analysis of cell viability as the percentage of DAPI^−^ cells revealed X-Vivo 15, SCGM, and StemSpan-ACF to be comparable, with the fraction of live cells exceeding 80% in all cases and with both donors (Figures S1B and S1C). Regarding cell maintenance and/or expansion, an initial drop in total cell counts was observed with these three media but recovered from day 2 onward, resulting in expansion (donor A) or maintenance (donor B) of total cell numbers (Figure 1B). No major differences between the media could be observed with donor B, and differences did not reach significance with donor A, yielding mean counts of 7.7 × 10^6^ cells with SCGM, 8.4 × 10^6^ cells with StemSpan-ACF, and 5.2 × 10^6^ cells with X-Vivo 15. Manual cell counts performed on day 3 demonstrated the three media to perform similarly, with SCGM and StemSpan-ACF yielding slightly higher cell numbers as compared to X-Vivo 15 (Figure S1D). Determination of the percentage of CD34^+^CD90^+^ HSPCs^prim^ within the cultures revealed an increase from day 0 to day 3 from 22.6% to 28.4% (X-Vivo 15), to 31.7% (SCGM), and to 27.4% (StemSpan-ACF) with donor A; and from 8.3% to 24.1% (X-Vivo 15), to 26.5% (SCGM), and to 23.4% (StemSpan-ACF) with donor B (Figure 1C). With that, SCGM appeared to be superior to the other media in terms of HSPC^prim^ percentage. HSPC^prim^ counts were maintained (X-Vivo 15, donor A) or expanded (X-Vivo 15, donor B; SCGM and StemSpan-ACF, both donors) over time (Figure S1E). No major differences were observed with both donors in terms of HSPC^prim^ counts in X-Vivo 15, SCGM, and StemSpan-ACF cultures. To assess the colony-forming potential of HSPCs after 3 days of culture in the different media, cells from donor B were subjected to *in vitro* colony-forming unit (CFU) assay. Manual count of colony types 2 weeks post-cell-seeding revealed no major differences in the numbers of burst-forming unit-erythroid (BFU-E), colony-forming unit-granulocyte macrophage (CFU-GM), and CFU-granulocyte, erythrocyte, monocyte, megakaryocyte (CFU-GEMM) (Figure 1D). In contrast to these three media, live cells were gradually lost upon culture in HSC Brew with both donors (Figure 1B; Figure S1C), and the HSPC^prim^ percentage and counts continuously decreased over time (Figure 1C; Figure S1E). Due to insufficient cell numbers, HSC Brew was excluded from manual counting and CFU analysis.

Altogether, SCGM was superior to X-Vivo 15 in terms of HSPC^prim^ percentage but not total cell counts and HSPC maintenance and/or expansion, while SCGM and StemSpan-ACF performed similarly. In contrast to StemSpan-ACF, SCGM that is manufactured to cGMP is currently available and is thus suitable for application in clinical protocols. Therefore, SCGM was selected for all further experiments.

### TEs Increase CD34^+^ HSPC Transduction Efficiency with LV and ARV Vectors

We selected 7 previously described TEs and systematically compared their effects on retroviral gene transfer and HSPC quality in SCGM. These included LentiBOOST,^29^ PGE2,^34^ PS,^30^ Vectofusin-1,^31^, ^32^, ^38^ ViraDuctin, RetroNectin,^28^ and Stauro^33^ (Figure S2A). Selection was also based on the availability of TEs manufactured to cGMP to allow for rapid translation of findings into clinical application. Taking this into consideration, we also included the synthetic, antineoplastic, clinically used 7-hydroxy derivative of Stauro (OH-Stauro), making 8 TEs in total. As OH-Stauro, to our knowledge, has not yet been tested as a TE, two different concentrations (referred to as “high” and “low”) were tested for OH-Stauro and for Stauro.

**Figure 2:**
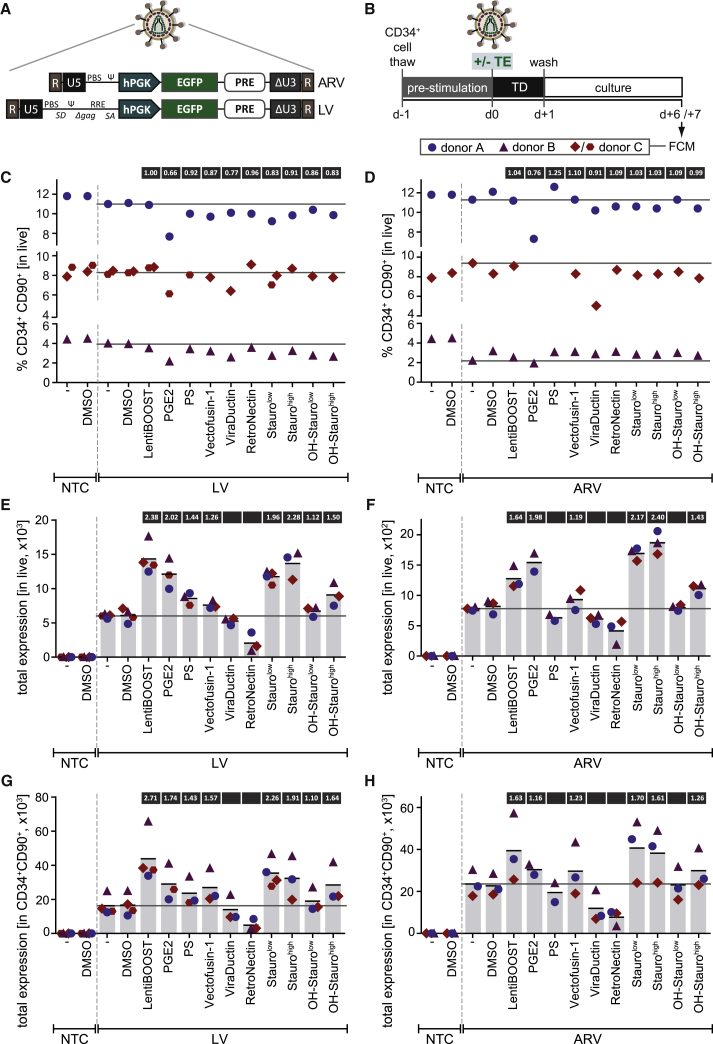
Single Transduction Enhancers Increase Transduction of CD34^+^ HSPCs with Lentiviral and Alpharetroviral Vectors (A) Schematic representation of the EGFP-encoding alpharetroviral (ARV) and lentiviral (LV) SIN vectors used in this study. R, repeat region; U5, unique 5; hPGK, human phosphoglycerate kinase promoter; PRE, post-transcriptional regulatory element; ΔU3, unique 3 region with self-inactivating deletion. (B) Experimental scheme. CD34^+^ HSPCs from 3 HDs were pre-stimulated for 24 h prior to transduction in the presence or absence of single transduction enhancers (TEs) in SCGM plus STF. Transduction was carried out at a MOI of 20, which is lower than the MOI used with most gene therapy protocols, allowing for a better observation of TE effects due to a lower transduction rate. Cells were washed after 24 h and analyzed by FCM 1 week post-transduction. Donor C was used in 2 independent experiments for selected conditions. (C and D) Percentage of CD34^+^CD90^+^ HSPCs^prim^ 1 week post-transduction with LV (C) and ARV (D) vectors at a MOI of 20. NTC, non-transduced control; -, no TE or vehicle; DMSO, vehicle only; PGE2, prostaglandin E2; PS, protamine sulfate; Stauro, staurosporine; OH-Stauro, 7-hydroxy-staurosporine. Horizontal lines indicate baseline levels in the absence of TE treatment (“-” condition) for each donor. (E and F) Total EGFP expression (= normalized percentage of EGFP^+^ cells × median EGFP intensity within the EGFP^+^ fraction) in live cells 1 week post-transduction with LV (E) and ARV (F) vectors at a MOI of 20. Vertical bars represent the mean total expression. Numbers in boxes above bars indicate the mean fold increase (if ≥1.1) from the four experiments. (G and H) Total EGFP expression in the CD34^+^CD90^+^ HSPC^prim^ fraction 1 week post-transduction with LV (G) and ARV (H) vectors at a MOI of 20. Vertical bars represent the mean total expression. Numbers in boxes above bars indicate the mean fold increase (if ≥1.1) from the four experiments.

While having been previously analyzed in combination with LV vectors, TE effects on ARV transduction efficiencies have not yet been determined. Due to the inherent safety features, which make them attractive for future clinical gene therapy, and the increasing preclinical use of ARV vectors for modification of HSPCs and other hematopoietic cell types,^24^, ^25^, ^26^, ^39^, ^40^, ^41^ we sought to identify compounds that enhance ARV gene transfer in parallel to testing TE effects on LV gene transfer. We used LV and ARV standard SIN EGFP reporter vectors with an identical design of the internal gene expression unit (Figure 2A). Purified CD34^+^ HSPCs from three HDs were transduced with VSV-G (vesicular stomatitis virus G protein)-pseudotyped vectors in the presence or absence of the selected TEs (Figure 2B). In order to investigate any effects on HSPC “stemness,” the percentage of CD34^+^CD90^+^ (HSPCs^prim^) was determined (Figures 2C and 2D; Figure S2B). Individual TEs had consistent effects regardless of their combination with LV or ARV vectors and could be grouped into two categories: (1) causing no (LentiBOOST and RetroNectin), or only a very mild reduction in the HSPC^prim^ percentage (PS, Vectofusin-1, Stauro, and OH-Stauro), and (2) causing a consistent reduction in the HSPC^prim^ percentage (PGE2 and ViraDuctin). PGE2 reduced the HSPC^prim^ percentage to an average of 66% (Figure 2C) and 76% (Figure 2D) of that of vehicle-only controls for LV and ARV transduction, respectively. Individual analysis of CD34 and CD90 antigen expression revealed the reduction in the HSPC^prim^ percentage to be almost exclusively resulting from a loss in CD90 expression in all cases (Figures S3A and S3B).

To determine TE effects on transduction efficiency, the percentage of EGFP^+^ cells was determined both in the live cell population and in the HSPC^prim^ fraction (Figures S2B and S3C–S3F). In the absence of TEs, LV and ARV transduction of HSPCs at a MOI of 20 yielded efficiencies of 38.7% (Figure S3C) and 9.0% (Figure S3D), respectively, revealing the LV vector to be more potent in our setting than its ARV counterpart. A preference for transduction of the HSPC^prim^ fraction was observed with the tested vector batches, reaching 57.0% (LV) and 17.7% (ARV) transduction efficiencies in the absence of TEs (Figures S3E and S3F). Of the 8 selected TEs, 6 increased LV and 5 elevated ARV transduction efficiencies (Figures S3C–S3F). However, with transduction efficiencies exceeding 30% and, thus, with the occurrence of multiple integrations in a substantial fraction of transduced cells, TE effects might be partially masked when analyzing %EGFP^+^. Therefore, we determined the median fluorescence intensity (MFI) of EGFP within the EGFP^+^ population and calculated total expression as the product of normalized %EGFP^+^ and MFI (Figures 2E–2H). This analysis confirmed that 6 of the tested TEs increased LV gene transfer to both the live cell (Figure 2E) and the HSPC^prim^ fraction (Figure 2G). The strongest effect was obtained with LentiBOOST, achieving a 2.4-fold increase in total expression within live cells and a 2.7-fold increase within HSPCs^prim^, followed by Stauro^low^ and Stauro^high^, PGE2, OH-Stauro^high^, PS and Vectofusin-1, and OH-Stauro^low^. In contrast, no positive effects were observed with ViraDuctin and RetroNectin. For transduction using ARV vectors, the same TEs showed benefit on gene transfer rates as for LV vectors, with the exception of PS, which increased total expression only with LV but not ARV vectors (Figures 2F and 2H). In the ARV setting, Stauro^high^ and Stauro^low^ performed best, increasing transduction by 2.4- and 2.2-fold, respectively, in the live cell (Figure 2F) and by 1.6- and 1.7-fold, respectively, in the HSPC^prim^ fraction (Figure 2H), followed by LentiBOOST, OH-Stauro^high^ and PGE2, and, finally, Vectofusin-1. No positive effects were seen with PS, ViraDuctin, RetroNectin, or OH-Stauro^low^.

Altogether, HSPC transduction with LV and ARV vectors was improved by more than 2-fold with the use of a single TE, the strongest effects having been observed with LentiBOOST, Stauro, and PGE2. Furthermore, the HSPC^prim^ percentage was not affected upon administration of LentiBOOST and only mildly decreased with Stauro treatment, while PGE2 supplement led to a pronounced reduction in HSPC^prim^ percentage.

### Combinatorial Use of Selected TEs Further Improves LV CD34^+^ HSPC Transduction

**Figure 3:**
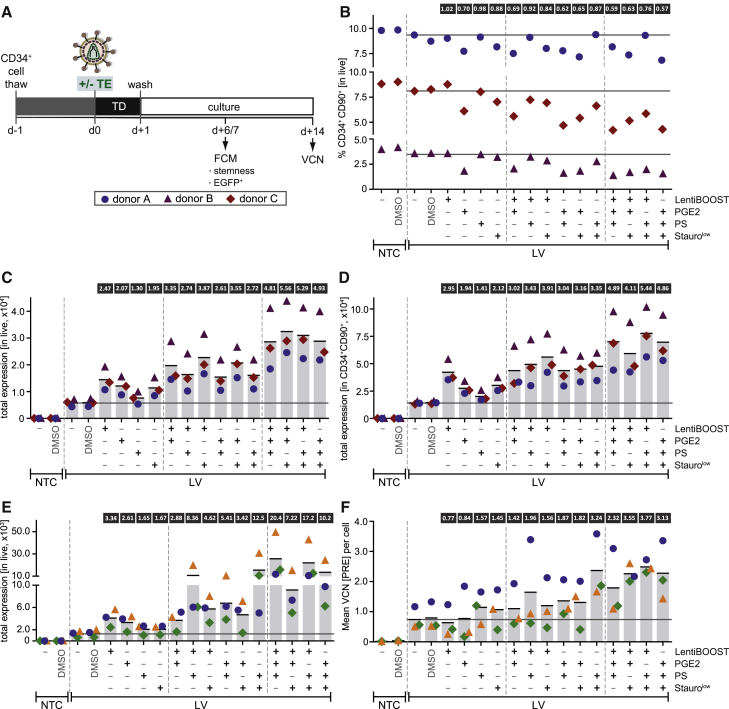
Combination of Transduction Enhancers Increases Lentiviral CD34^+^ HSPC Transduction over Their Single Use (A) Experimental timeline. CD34^+^ HSPCs from 3 HDs were pre-stimulated for 24 h and then transduced with the LV vector depicted in Figure 2A at MOIs of 20 and 10 in the presence or absence of selected transduction enhancer (TE) combinations in two sets of experiments. Cells were cultured in SCGM plus STF. Cells were washed after 24 h and analyzed by FCM 1 week post-transduction. Vector copy numbers (VCNs) were determined 2 weeks post-transduction (MOI 10 set of experiments). (B) Percentage of CD34^+^CD90^+^ HSPCs^prim^ 1 week after LV transduction at a MOI of 20. NTC, non-transduced control; -, no TE or vehicle; DMSO, vehicle only; PGE2, prostaglandin E2; PS, protamine sulfate; Stauro, staurosporine. Horizontal lines indicate baseline levels in the absence of TE treatment (“-” condition) for each donor. Numbers in boxes above the graph indicate the mean fold change from the three experiments, relative to the “no TE” transduced control. (C and D) Total EGFP expression (= normalized percentage of EGFP^+^ cells × median EGFP intensity within the EGFP^+^ fraction) in the live (C) and in the CD34^+^CD90^+^ HSPC^prim^ (D) fraction 1 week after LV HSPC transduction at a MOI of 20. Vertical bars represent the mean total expression. Numbers in boxes above bars indicate the mean fold increase (if ≥1.1) from the three experiments. (E) Total EGFP expression in live cells 1 week post-LV-HSPC-transduction at a MOI of 10. Vertical bars represent the mean total expression. Numbers above bars indicate the mean fold increase (if ≥1.1) from the three experiments. Note: for 2 (green and orange symbols) of the 3 donors, Stauro treatment was performed for 24 h, including the transduction period, instead of the 2-h pre-incubation period chosen in previous experiments. (F) Vector copy number (VCN) per diploid cell, determined by real-time qPCR detecting the post-transcriptional regulatory element (PRE) 2 weeks post-transduction at a MOI of 10. Vertical bars represent the mean of three experiments. Numbers in boxes above bars indicate the mean fold increase. Note that, for 2 (green and orange symbols) of the 3 donors, Stauro treatment was performed for 24 h, including the transduction period, instead of the 2-h pre-incubation period chosen in previous experiments.

We next investigated whether the combinatorial use of TEs would further improve transduction, potentially yielding additive or synergistic effects. As LV, in contrast to ARV, vectors are already clinically applied, we focused on LV HSPC transduction in this set of experiments. The four best performing TEs—LentiBOOST, PGE2, PS, and Stauro^low^—were selected and co-administered in groups of two or three in all possible combinations. Purified CD34^+^ HSPCs from three HDs were transduced with the VSV-G-pseudotyped, EGFP-expressing LV vector as shown in Figure 2A. Transduction was performed at a MOI of 20 in SCGM in the absence or presence of single TEs or their combinations (Figure 3A).

The percentage of CD34^+^CD90^+^ (HSPCs^prim^) was used to determine “stemness” 1 week post-transduction. As a general observation, the HSPC^prim^ percentage gradually decreased with increasing numbers of TEs combined together (Figure 3B). However, the decrease in “stemness” upon combination of LentiBOOST, PS, and Stauro^low^ was mild, reaching levels of >80% (two TEs) and of 76% (three TEs) of that of a no-TE-control. LentiBOOST plus PS performed best for two TEs combined, with the HSPC^prim^ percentage constituting 92% that of the non-treated control. In contrast, as with single use, all combinations including PGE2 reduced the HSPC^prim^ percentage more than TE cocktails without PGE2, namely, to 62%–69% (two TEs) and to 57%–63% (three TEs). Again, the decrease in the HSPC^prim^ percentage was reflected in a reduced CD90 antigen expression in all cases (Figure S4A).

Transduction efficiencies, determined as %EGFP^+^ 1 week post-transduction, reached 38.8% in live cells in the absence of TEs and was increased to 47%–69% with single-TE treatment, to 68%–83% with two TEs, and to even 84%–91% with three TEs (Figure S4B), indicating that TE combination provides additional benefit. Within the CD34^+^CD90^+^ HSPC^prim^ fraction, transduction efficiencies were close to saturation with two TEs and could not be further increased with three TEs (Figure S4C). As for the single-TE analysis, total expression was calculated and displayed stronger effects. In live cells, combinatorial use of two TEs yielded a 2.61- to 3.87-fold increase, and combinatorial use of three TEs yielded a 4.81- to 5.56-fold increase in total expression (Figure 3C). Within the HSPC^prim^ fraction, LentiBOOST plus PS and LentiBOOST plus Stauro^low^ were the most efficient among the combinations of two TEs, and the combination of these compounds (LentiBOOST plus PS plus Stauro^low^) yielded the highest increase in total expression among the groups of three TEs (Figure 3D).

With transduction levels close to saturation at a MOI of 20, a second set of experiments was performed using a lower MOI of 10 to further differentiate TE effects. Vector copy number (VCN) analysis was included as a second readout, performed on day 14 post-transduction to allow for non-integrated episomal vector copies to be diluted out prior to analysis (Figure 3A). Transduction efficiencies in live cells could be increased from 8.8% in the absence of TE treatment to up to 65.7% with three TEs (Figure S4D). As expected, observed TE effects on %EGFP^+^ (Figure S4D) and on total expression (Figure 3E) were much more pronounced in the low-MOI setting. LentiBOOST plus PS (8.36-fold increase) and PS plus Stauro^low^ (12.5-fold increase) were most potent among the combinations of two TEs, and LentiBOOST plus PGE2 plus PS (20.4-fold increase) was superior to all others. When analyzing VCNs, an increase of 1.42- to 3.24-fold was observed with two TEs, and an increase of 2.32- to 3.77-fold was observed with three TEs (Figure 3F). VCN analysis mostly reflected the effects seen with total expression analysis, confirming LentiBOOST plus PS and PS plus Stauro^low^ as the most efficient combinations of two TEs. Interestingly, the single use of LentiBOOST and PGE2 did not increase VCNs, although they achieved the strongest effects in terms of total expression among the four tested compounds. In turn, while PS and Stauro^low^ achieved less pronounced effects with regard to total expression upon single use, they increased VCNs by 1.57- and 1.45-fold, respectively.

### Combinatorial Use of LentiBOOST and PS Does Not Cause Major Alterations in HSPC Gene Expression Profiles

To investigate TE effects on HSPC quality and potential toxicity in more detail by assessing potential alterations in the transcriptional activity of HSPCs after viral transduction and TE addition, we performed whole transcriptome analysis. LentiBOOST and PS were selected as the best performing and, thus, most attractive TE combination for clinical use based on (1) total expression in the HSPC^prim^ fraction upon transduction at a MOI of 20, (2) total expression in live cells upon transduction at a MOI of 10, (3) VCNs upon transduction at a MOI of 10, and (4) HSPC^prim^ percentage upon transduction of non-clinical EGFP-reporter vectors. To test the effect of these two TEs on gene expression profiles, purified CD34^+^ HSPCs from three HDs were sorted for the CD34^+^CD38^−^ population directly after the cell thaw to enrich for the stem cell fraction (Figures S5A and S5B). CD38 was used as a marker, as cells were immediately characterized without any prior culturing and, thus, without the risk of false-CD38-negatives arising as an artifact of prolonged culture. The percentage of CD34^+^CD38^−^ cells constituted 14.9%–26.7% of the total population in all three donors (Figure S5C). After a 24-h pre-stimulation period, transduction was accomplished using the LV EGFP vector, shown in Figure 2A, at a MOI of 20 in the presence or absence of LentiBOOST plus PS. The sorted cells from each donor were split into the following four conditions: (1) negative control (untreated; NTC), (2) TE control (TE supplement without LV transduction), (3) transduction control (LV; transduction in the absence of TEs), and (4) LV vector transduction in the presence of TEs (LV+TE). The day after transduction, samples were EGFP sorted, and RNA was harvested. As before, TE treatment prominently improved LV HSPC transduction, elevating the percentage of EGFP^+^ cells from 61.4% to 98.8% upon TE administration averaged across all three donors (Figures S5D and S5E).

**Figure 4:**
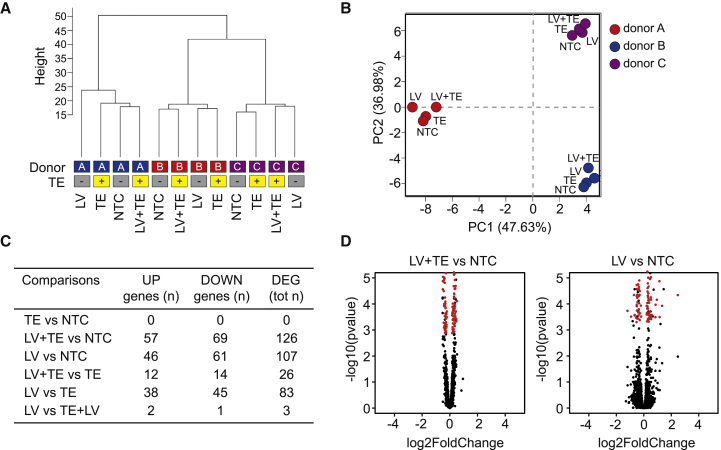
LentiBOOST Plus Protamine Sulfate Do Not Cause Major Alterations in HSPC Gene Expression Profiles (A) Hierarchical clustering of RNA-seq data. NTC, non-transduced and non-TE-treated control; TE, non-transduced control treated with TE only; LV, transduced in the absence of TE; LV+TE, transduced in the presence of TE. (B) First two components of a principal-component analysis, with percentage of variance associated with each axis; each dot represents a sample, colored according to donor source. (C) List of differentially expressed genes (DEGs) found to be significantly deregulated (adjusted p ≤ 0.05) for each treatment comparison. (D) Volcano plots of RNA-seq data from three biological replicates; differentially expressed transcripts (adjusted p ≤ 0.05) between LV-treated or LV+TE-treated and untreated HSPCs are highlighted in red.

An unsupervised cluster analysis of the RNA-sequencing (RNA-seq) samples identified three main branches corresponding to the different donor cell sources (Figure 4A). Within each branch, LV-treated or LV+TE-treated samples did not form distinct clusters, compared with untreated samples, indicating substantial overlap between the gene expression profiles of treated and untreated samples. Principal-component analysis confirmed these results, with donor variability being the main source of variance in the data (Figure 4B). Using a supervised hierarchical ordering approach, we identified 107 and 126 genes that varied significantly (p ≤ 0.05) between untreated and LV- or LV+TE-treated samples, respectively (Figure 4C). However, when analyzing the extent of expression changes, 0 or 19 genes were differentially expressed by ≥1.5-fold in LV+TE- or LV-treated samples compared to untreated samples (Figure 4D; Figure S6). Moreover, Gene Ontology analysis of differentially expressed genes showed no enrichment for functional categories, suggesting that the modest transcriptional changes seen upon HSPC transduction with or without TE do not reflect a functional up- or downregulation of specific cellular pathways. Such findings are in keeping with the absence of sample segregation according to transduction or TE addition, as observed by the unsupervised clustering analysis, and indicate that our gene therapy protocol causes no major alterations in the gene expression program of healthy HSPCs.

### Combination of LentiBOOST and PS Increases CD34^+^ HSPC Gene Transfer Using a Clinical LV Vector under GMP Conditions

**Figure 5:**
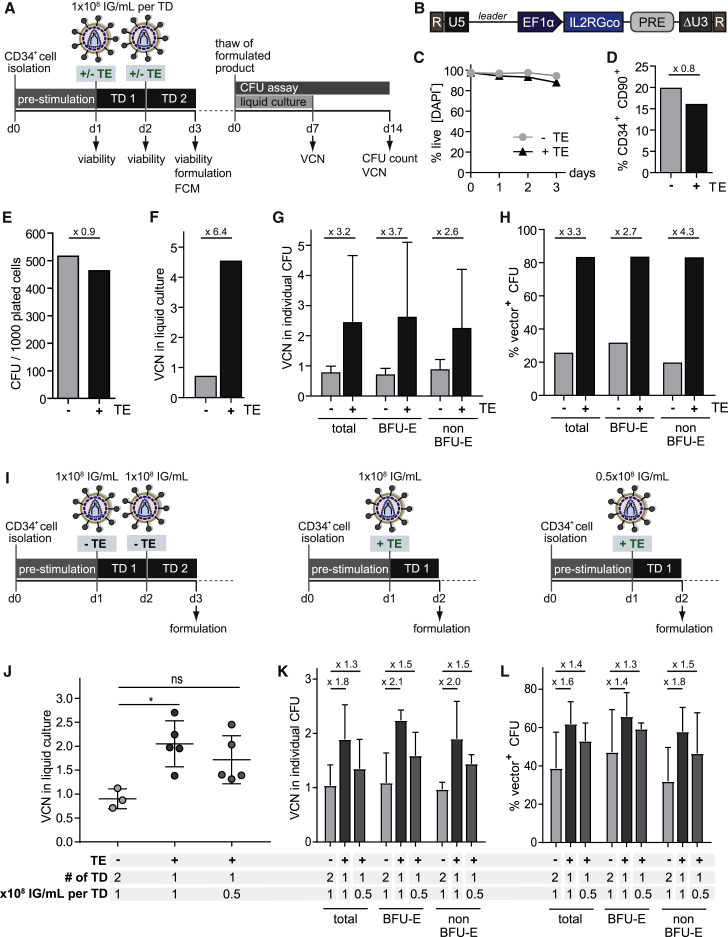
LentiBOOST Plus Protamine Sulfate Increase Transduction of CD34^+^ HSPCs with a Clinical-Grade Lentiviral SCID-X1 Vector under GMP Conditions (A) Experimental timeline. Fresh CD34^+^ HSPCs were purified from a mobilized leukapheresis and pre-stimulated for 24 h in SCGM supplemented with STF. Transduction was performed at a MOI of 66.66 (1 × 10^8^ IG/mL) in the presence or absence of the TEs LentiBOOST and PS in two rounds on consecutive days. Viability was determined regularly by trypan blue staining and manual cell count. The product was formulated on day 3 post-selection, and cells were analyzed for the expression of stem cell markers by FCM. An aliquot of the formulated product was thawed and analyzed for the VCN 1 week after liquid culture and for CFUs, as well as for the VCN in individual colonies, after 2 weeks of culture in MethoCult (CFU assay). All steps were performed under GMP conditions and according to GMP protocols. (B) Schematic representation of the LV vector clinically used for the treatment of SCID-X1, with a SIN design to express the interleukin-2 receptor gamma chain (IL2RG). R, repeat region; U5, unique 5; EF1α, human elongation factor 1α promoter; PRE, post-transcriptional regulatory element; ΔU3, unique 3 region with self-inactivating deletion. (C) Percentage of live cells, determined by trypan blue exclusion. −TE, no transduction enhancer; +TE, supplemented with LentiBOOST and PS. (D) Percentage of CD34^+^CD90^+^ HSPCs^prim^ on day 3 post-thaw. −TE, no transduction enhancer; +TE, supplemented with LentiBOOST and PS. (E) CFU assay. Individual colony types were manually counted after 2 weeks of culture in MethoCult. −TE, no transduction enhancer; +TE, supplemented with LentiBOOST and PS. (F) Mean VCN of the LV Psi sequence per diploid cell, determined by real-time qPCR after 1 week of liquid culture. −TE, no transduction enhancer; +TE, supplemented with LentiBOOST and PS. (G) VCN (Psi) in individual CFU colonies. Error bars indicate the mean ± SD. −TE, no transduction enhancer; +TE, supplemented with LentiBOOST and PS. (H) Percentage of vector-positive CFUs in different CFU fractions, determined by real-time qPCR detecting the Psi sequence. (I) The clinical transduction protocol involving two rounds of transduction at 1 × 10^8^ IG/mL in the absence of TE administration was tested against two modified protocols entailing the use of the TEs LentiBOOST and PS, i.e., only one round of transduction at the full vector dose or at half the original vector dose (0.5 × 10^8^ IG/mL). VCN determination in liquid culture and CFU assays were performed as shown in Figure 5A. −TE, no transduction enhancer; +TE, supplemented with LentiBOOST and PS. (J) Mean VCNs of the LV Psi sequence per diploid cell, determined by real-time qPCR after 1 week of liquid culture. *p ≤ 0.05, Kruskal-Wallis test with Dunn’s multiple comparison. Bars indicate the mean ± SD. (K) VCNs (Psi) in individual CFU colonies. Error bars indicate the mean ± SD. (L) Percentage of vector-positive CFUs in different CFU fractions, determined by real-time qPCR detecting the Psi sequence. Error bars indicate the mean ± SD.

We next sought to validate the identified culture and transduction conditions in a clinically relevant setting. Thus, selected conditions were applied in a GMP-compliant ATMP manufacturing process for SCID-X1.^42^ Freshly purified CD34^+^ HSPCs were transduced in SCGM with SCID-X1 LV vector in accordance with our clinical protocol (Figures 5A and 5B).^42^ LentiBOOST and PS were added during both transductions and compared to a non-TE-treated control.

Viability was mildly reduced in the TE-treated culture, as compared to the control culture, especially on day 3 of the protocol (Figure 5C). However, day-3 viability within the TE-treated culture was still acceptable, constituting 87.9%. FCM performed on day 3 of the procedure identified 19.8% of CD34^+^CD90^+^ HSPCs^prim^ for the control culture and 16.0% of HSPCs^prim^ for the TE-treated sample (Figure 5D). The capacity for *in vitro* proliferation and differentiation was determined by standard CFU assay (Figure 5A), revealing the control culture to give a slightly higher number of colonies than the TE-treated sample (Figure 5E). In order to determine the effects of TEs on transduction rates, VCNs were assessed through real-time qPCR. After 7 days of liquid culture (Figure 5A), mean VCNs per cell of 0.71 and of 4.53 were detected for the control and the TE-treated samples, respectively, revealing a 6.4-fold increase upon TE administration (Figure 5F). VCN analysis also showed an increase of 3.2-fold in individual CFU colonies upon TE treatment, and this increase was consistent across different colony types (total CFU, BFU-E, and non-BFU-E) (Figure 5G). Similarly, a 3.4-fold increase upon TE treatment was observed in the percentage of vector-positive CFUs (Figure 5H).

The observed increase in the VCN upon TE addition offers the potential to reduce vector dose or the number of transductions required to achieve sufficient transduction rates. Using the clinical-grade SCID-X1 vector batch as before, we tested two further protocols incorporating the TEs LentiBOOST and PS: (1) only one round of transduction at the full dose of 1 × 10^8^ infectious genomes (IG)/mL and (2) one round of transduction at half the dose, i.e., at 0.5 × 10^8^ IG/mL (Figure 5I). In contrast to the previous protocol, the final product is formulated 1 day earlier. VCNs in liquid cultures were significantly higher (one transduction at the full dose) or comparable (one transduction at half the original dose) when compared to the conventional protocol using two rounds of transduction at 1 × 10^8^ IG/mL in the absence of TE (Figure 5J), showing that TE addition allows for a reduction in vector dose to one fourth the original amount. In individual CFUs, VCNs were in a range of between 0.7 and 2.7 copies per cell and were higher upon TE addition at half and at one fourth of the total vector amount, as compared to the full vector dose applied in the absence of TE (Figure 5K). Similarly, an increased percentage of vector-positive CFUs was observed upon TE administration at reduced total vector doses, as compared to the conventional transduction protocol (Figure 5L).

## Discussion

Genetic modification of CD34^+^ HSPCs offers a cure for monogenic hematological disorders. Despite initial success in a number of disease contexts, relatively low permissiveness of CD34^+^ HSPCs to gene transfer, patient-to-patient variability, and difficult-to-treat disease contexts require the identification of strategies to overcome existing barriers and to optimize current procedures toward reliable, context- and patient-independent, and cost-effective treatment protocols. Innate barriers limiting gene transfer into HSPCs impose the use of high MOIs in clinical procedures, which, in most cases, also entails multiple rounds of viral vector administration and prolonged *ex vivo* culture. In this study, we addressed both the aspect of defining suitable *ex vivo* culture conditions and the aspect of increasing retroviral gene transfer into CD34^+^ HSPCs by conducting a systematic comparison of culture media and TEs under clinically relevant conditions.

An optimal HSPC culture medium is serum-free and completely defined, is manufactured to cGMP, and maintains or expands cell numbers while preserving HSPCs. In a side-by-side comparison, we revealed SCGM to support the greatest expansion in HSPC^prim^ percentage. Using our protocol, SCGM plus hSTF (human stem cell factor (hSCF), human thrombopoietin (hTPO), and human Flt3-Ligand (hFLT3-L) cytokines applied at clinically used concentrations also allowed efficient LV transduction of CD34^+^ HSPCs, with around 40% of transduced cells at a MOI of 20. Interestingly, ARV transduction efficiencies were below 10% when applied at the same MOI, suggesting that, although potentially superior in terms of integration safety profiles, ARV vectors may be less efficient in CD34^+^ HSPC transduction than LV counterparts.

Use of single TEs potently increased both LV and ARV HSPC transduction. All compounds, except for OH-Stauro (not previously tested), had been previously identified to improve LV CD34^+^ HSPC transduction and were selected based on these positive results.^27^, ^28^, ^29^, ^30^, ^31^, ^32^, ^33^, ^34^ Optimally, to be included in clinical procedures, a TE fulfills the following characteristics: (1) absence of cytotoxicity and preservation of stem cell quality, (2) positive influence on transduction, (3) availability as manufactured to cGMP product, and (4) easy application. While PGE2 and ViraDuctin treatment lowered the HSPC^prim^ frequency according to surface markers, and ViraDuctin as well as RetroNectin administration did not achieve improved LV HSPC transduction, LentiBOOST, PS, Vectofusin-1, Stauro, and OH-Stauro all elevated transduction efficiencies with minimal detrimental effect on HSPC^prim^ content. The greatest increase in LV HSPC transduction was observed with LentiBOOST. As most TEs act on the attachment or entry process, they were likely to be applicable to boost not only LV transduction but also that of other retroviral family members when using the same entry route or envelope. Indeed, 4 of the 5 TEs enhancing LV transduction also increased ARV gene transfer. Interestingly, and in contrast to all other TEs, PS only stimulated LV but not ARV HSPC transduction, although both particle types were pseudotyped with VSV-G, suggesting that vector- or vector-genus-intrinsic characteristics might also play a role in this context.

Dissecting the individual effects, we could observe preferential effects for some of the TEs included in our panel on one or several of the following parameters: (1) transduction efficiency, (2) VCN, or (3) expression level (MFI). LentiBOOST and PGE2 both yielded a strong increase in transduction efficiency, while VCNs were not enhanced. This suggests that, rather than achieving an absolute enhancement in transduction, these compounds affect the distribution of vectors in the cell population, yielding more vector-positive cells but with less vector copies per cell. An unaltered VCN upon LentiBOOST-assisted HSPC transduction was likewise observed by others when applied at similar concentrations.^29^ We also observed an increased MFI of EGFP within the EGFP^+^ population (data not shown, but reflected by the total expression) upon administration of LentiBOOST, a putative entry enhancer. To achieve an increased MFI in the absence of an increased VCN, we hypothesize that LentiBOOST has additional effects beyond the enhancement of fusion between vector particles and cells that might play a role in elevated expression levels. In that regard, it would be interesting to compare integration patterns and explore entry pathways and intracellular trafficking routes, as well as the accessibility of LV particles to restriction factors in the presence and absence of LentiBOOST. Another possible explanation for the differential effect of LentiBOOST on total expression (determined 1 week post-transduction) and VCNs (determined 2 weeks post-transduction) is that it, in a transient manner, facilitates vector entry in the absence of successful integration, with the vector in the form of extrachromosomal episomes having been diluted out through cell division prior to VCN analysis. Determination of transduction efficiency at later time points could shed more light on this aspect. Nevertheless, even at 2 weeks post-transduction, LentiBOOST increased VCNs in combination with PS over the numbers achieved with PS alone. Furthermore, Hauber et al.^29^ demonstrated a consistent increase in LV CD34^+^ cell transduction efficiency at different time points, including day 12 post-transduction, upon LentiBOOST supplement, here also in the absence of an increased VCN, pointing to an altered vector distribution rather than an only transient effect based on facilitated vector entry without integration.

For clinical gene therapy protocols, it may be beneficial to combine TEs that differentially affect individual transduction parameters in order to achieve a balanced increase in both transduction efficiency across the whole cell population and VCN, while avoiding excessively high VCNs. Furthermore, exerting different mechanisms of action to overcome innate barriers and enhance transduction, TEs might also mechanistically complement each other, opening up the possibility to achieve even greater effects upon their combination. The selected TEs in this study operate through different mechanisms of action: PS and ViraDuctin lower the electrostatic repulsion between particles and target cell membranes;^30^ RetroNectin physically increases co-localization of vector particles and target cells;^27^ PGE2 acts intracellularly by enhancing an as-yet uncharacterized post-entry step;^34^ Stauro, as a serine/threonine kinase inhibitor, enhances cell entry by stimulating actin dynamics;^33^ Vectofusin-1 is an amphipathic peptide helix that promotes both adhesion and fusion between viral particles and target cells;^31^, ^32^, ^43^ and LentiBOOST is hypothesized to act as an entry enhancer.^29^ The co-administration of TEs has been previously tested in a very limited number of combinations,^29^, ^33^, ^44^ and a systematic comparison of different combinations and their effects has, so far, not been reported. The combination of Stauro and PGE2 has been described to further increase the transduction efficiency as compared to either compound alone; however, effects on VCNs diminished in long-term NSG transplants.^33^ Co-administration of LentiBOOST and PS did not increase transduction efficiency over the use of LentiBOOST alone; however, with efficiencies above 80%, this might have been due to saturation, and VCNs were, indeed, further increased upon PS addition.^29^ For systematic, rational combination of TEs in groups of two or three, we chose compounds that had yielded the strongest effects on LV HSPC transduction on their single use and that could be expected to mechanistically complement each other, including LentiBOOST, PGE2, PS, and Stauro. Indeed, all combinations increased the magnitude of effects over their single use, with a maximum effect in the combinations of two TEs achieved with LentiBOOST plus PS and with PS plus Stauro. All three compounds are available as manufactured to cGMP products and are part of clinical protocols already, making these combinations attractive for immediate clinical translation. In our hands, the combination of LentiBOOST and PS was slightly superior to all others in terms of preserving HSPCs^prim^ according to surface markers. Therefore, LentiBOOST and PS were chosen for the manufacturing of an ATMP for SCID-X1 and demonstrated a 6.4-fold increase in VCN. Furthermore, combination of these TEs allowed for a reduction of the original vector dose by one half and a reduction of the number of transductions from two rounds to one (i.e., using, in total, one fourth of the original vector dose) while still yielding increased VCNs. On this basis, treatment costs for future clinical gene therapy approaches could be lowered, and *ex vivo* HSPC culture time be reduced, which is also likely to positively benefit HSPC quality.

When using reagents and excipients for the GMP manufacture of an ATMP, it is important to consider the standards met by them and their availability: PS can be easily obtained as a licensed medicinal product for human use. LentiBOOST is supplied by SIRION Biotech. For commercial and clinical use, SIRION licenses the technology. For non-profit research and clinical development in phase I/II, SIRION provides a royalty-free license to its technology.

We have defined optimal HSPC culture conditions and provide insights into individual TE effects. These may serve as a guideline for the rational, context-dependent choice of TEs or their combination to be included in clinical gene therapy protocols to increase treatment efficacy, to reduce patient-to-patient variability, to lower treatment cost, and, with that, to contribute to the overall success of clinical gene therapy.

## Materials and Methods

### Cell Culture

Primary human CD34^+^ HSPCs were seeded at 1 × 10^6^ cells per milliliter in culture well plates in GMP SCGM (CellGenix, Freiburg, Germany) supplemented with 1 U/mL penicillin plus 100 μg/mL streptomycin (GIBCO, Thermo Fisher Scientific, Rochford, UK), 300 ng/mL hSCF, 300 ng/mL hFlt3-L, and 100ng/mL hTPO (either manufactured to cGMP from CellGenix or animal-free from PeproTech, London, UK), referred to as STF.^45^ For comparison of HSPC culture media, using the same supplements as described earlier for SCGM, cells were cultured in X-Vivo 15 (Lonza, Slough, UK) additionally supplemented with 1% human albumin (Zenalb 20, Bio Products Laboratory, Herts, UK), in StemSpan-ACF (STEMCELL Technologies, London, UK) additionally supplemented with 1% human albumin, or in HSC Brew GMP medium plus HSC Brew GMP supplement (Miltenyi Biotec, Surrey, UK) additionally supplemented with 2% human albumin.

For the GMP experiment, HD CD34^+^ HSPCs were isolated from a fresh mobilized leukapheresis (AllCells, Alameda, CA, USA) following standard immunomagnetic procedure (CliniMACS, Miltenyi Biotec). Cells were seeded at 1–1.5 × 10^6^ cells per milliliter in SCGM supplemented with 300 ng/mL hSCF, 300 ng/mL hFlt3-L, and 100 ng/mL hTPO (manufactured to cGMP from CellGenix). Cells were cultured in VueLife Culture Bags (CellGenix).

### CD34^+^ HSPC Transduction

The TE concentrations used were as recommended in previous reports: LentiBOOST, 1 mg/mL (SIRION Biotech, Planegg, Germany); PGE2, 10 μM (Cayman Chemical, Cambridge, UK); PS, 4 μg/mL (Thermo Fisher Scientific); Vectofusin-1, 10 μg/mL (Miltenyi Biotec); ViraDuctin, 1× (Cambridge Bioscience, Cambridge, UK); RetroNectin, 20 μg/mL (TaKaRa Bio Europe, Saint-Germain-en-Laye, France); and Stauro (Cambridge Bioscience)/7-hydroxy-stauro (MERCK Chemicals, Watford, UK), 400 nM and 800 nM. For small-scale TE testing, 1 × 10^5^ cells were transduced at 1 × 10^6^ cells per milliliter in 96-well round-bottom plates (100 μL per well) at a MOI of 20 or of 10. For LentiBOOST-, PGE2-, and PS-assisted transduction, cells were pelleted and resuspended in the pre-mixed transduction cocktail composed of complete medium, TE, and viral vector. Vectofusin-1 and ViraDuctin were used as per manufacturer’s instructions. For RetroNectin-based transduction, 48-well plates were coated for 2 h with 4 μg RetroNectin per well (200 μL of a 20 μg/mL stock) and blocked for 30 min at room temperature with 2% BSA (Sigma-Aldrich, Poole, UK) in PBS (GIBCO). Plates were washed with Hank’s balanced salt solution (GIBCO) supplemented with 2.5% of 1 M HEPES (GIBCO). Viral vector in a total volume of 150 μL per well was added, and plates were centrifuged for 2 h at 32°C and 1,000 × *g*. After centrifugation, the viral-vector-containing supernatant was removed, and 1 × 10^5^ cells were added in 200 μL complete medium. For stauro- and 7-hydroxy-stauro-assisted transduction, cells were pre-incubated with the compounds for 2 h at 37°C. Cells were then washed in SCGM supplemented with penicillin/streptomycin, pelleted for 5 min at 300 × *g*, and resuspended in transduction cocktail consisting of complete SCGM and viral vector. For all protocols, cells were washed 16–24 h post-transduction and resuspended in fresh complete SCGM.

For the GMP experiment, cells were transduced two consecutive times after 24 h pre-stimulation. The vector was added to the media at a concentration of 10^8^ IG/mL, leading to a MOI of 66.6. The vector that was used, CCL_pEF1a_IL2RGcoWPRE*, was manufactured to cGMP (Yposkesi, Corbeil-Essonnes, France).^42^ Transduction was conducted either in the absence or presence of LentiBOOST and PS at the concentrations stated earlier. After each transduction cycle (16–18 h), cells were washed and resuspended in fresh complete SCGM. After the second transduction, cells were formulated in a commercial freezing mix containing 5% DMSO (CryoStor CS5, BioLife Solutions, Bothell, WA, USA) and cryopreserved using a control rate freezer. All steps were performed according to GMP guidelines and using GMP-approved materials and procedures.

### Flow Cytometry

Cells were harvested, washed, and centrifuged for 5 min at 300 × *g*. Pellets were resuspended in sterile-filtered FCM buffer composed of PBS supplemented with 0.5% BSA and 2 mM EDTA (Invitrogen, Paisley, UK). Cells were recorded on the BD LSR II flow cytometer (BD Biosciences, Oxford, UK) using BD FACSDiva software, and data were analyzed using FlowJo software (TreeStar., Ashland, OR, USA). The following antibody panel was used for HSPC^prim^ characterization: APC Mouse anti-CD90 (eBioscience, Thermo Fisher Scientific, catalog #17-0909-42) and BV421 Mouse anti-Human CD34 Clone 581 (RUO) (BD Horizon, BD Biosciences, catalog #562577). Cell counts were determined using CountBright Absolute Counting Beads (Invitrogen). Live and dead cells were discriminated using 1 μg/mL DAPI (Sigma-Aldrich). The percentage of HSPCs^prim^ was determined within the live-cell fraction in all cases.

For enrichment of CD34^+^CD38^−^ HSPCs, cells were stained with BV421 Mouse anti-Human CD34 Clone 581 (RUO) (BD Horizon, catalog #562577) and APC/Cy7 Mouse anti-Human CD38 Clone HIT2 (BioLegend, catalog #303534, London, UK) and sorted on a FACSAria III (BD Biosciences) cell sorter.

### RNA-Seq

Total RNA was isolated using TRIzol (Invitrogen) and the RNeasy Mini Kit (QIAGEN, Crawley, UK). RNA-seq libraries were prepared from 50 ng RNA using the NEBNext Single Cell/Low Input RNA Library Prep Kit for Illumina (New England Biolabs, Hitchin, UK), and 75-bp single-end sequences were obtained on a NextSeq 500 Instrument (Illumina, Cambridge, UK). Sequence tags were mapped to reference genome Hg19 using STAR aligner v2.5.3a,^46^ and raw read counts from HTSeq^47^ were used to estimate transcript levels. Unsupervised principal component analysis was performed on log_2_-transformed normalized counts using R (https://www.r-project.org). We then used DESeq2^48^ to normalize data using size factors or rlog transformation and to identify significant (adjusted p value ≤ 0.05) differential expression. Functional cluster analysis of differentially expressed genes was performed using Ingenuity Pathways Analysis (Ingenuity Systems) to identify the most relevant molecular interactions, functions, and pathways linking them. Sequencing files have been deposited into the GEO public database under accession number GEO: GSE129386.

### Statistics

Columns represent the mean of different replicates. Fold induction was calculated in relation to a non-TE-treated, transduced control. Fold induction was first calculated individually for each donor setting and then expressed as the mean of the individual fold induction values. Error bars represent the mean ± SD. Statistical testing was accomplished using GraphPad Prism software (GraphPad Software, San Diego, CA, USA) and two-way ANOVA with Geisser-Greenhouse correction (*p ≤ 0.05) or Kruskal-Wallis test with Dunn’s multiple comparison (*p ≤ 0.05).

### Ethics Statement

For usage of human CD34^+^ HSPCs from HDs, informed written consent was obtained in accordance with the Declaration of Helsinki and ethical approval from the Great Ormond Street Hospital for Children NHS Foundation Trust and the Institute of Child Health Research Ethics (08/H0713/87).

Information about vector cloning and production, VCN determination, and CFU assay can be found in Supplemental Materials and Methods.

## Author Contributions

J.W.S. designed and conducted experiments, analyzed and illustrated data, and wrote the manuscript. D.L.-R. initiated the study, performed experiments, and reviewed the manuscript. C.B.F. performed experiments and reviewed the manuscript. A.C. performed RNA-seq analysis and reviewed the manuscript. K.F.B. performed experiments and reviewed the manuscript. G.S. helped with experimental design. M.A.A. determined VCNs in CFU colonies. A.S. contributed vector technology and reviewed the manuscript. A.J.T. initiated the study and reviewed the manuscript.

## Conflicts of Interest

A.J.T. is on the scientific advisory boards of Orchard Therapeutics and Rocket Pharmaceuticals. The remaining authors declare no conflicts of interest.

## Supplemental Information

Document S1. Figures S1–S6 and Supplemental Materials and Methods

Document S2. Article plus Supplemental Information
