# Supplementary material for: Enhancing Lentiviral and Alpharetroviral Transduction of Human Hematopoietic Stem Cells for Clinical Application
Source: Mol Ther Methods Clin Dev. 2019 Jun 7;14:134–47. doi: 10.1016/j.omtm.2019.05.015 (PMC6629974; doi:10.1016/j.omtm.2019.05.015)
Supplement: Document S1. Figures S1–S6 and Supplemental Materials and Methods [file mmc1.pdf]

OMTM, Volume *14*

Supplemental Information

Enhancing Lentiviral and Alpharetroviral Transduction of Human Hematopoietic Stem Cells for Clinical Application

Juliane W. Schott, Diego León-Rico, Carolina B. Ferreira, Karen F. Buckland, Giorgia Santilli, Myriam A. Armant, Axel Schambach, Alessia Cavazza, and Adrian J. Thrasher

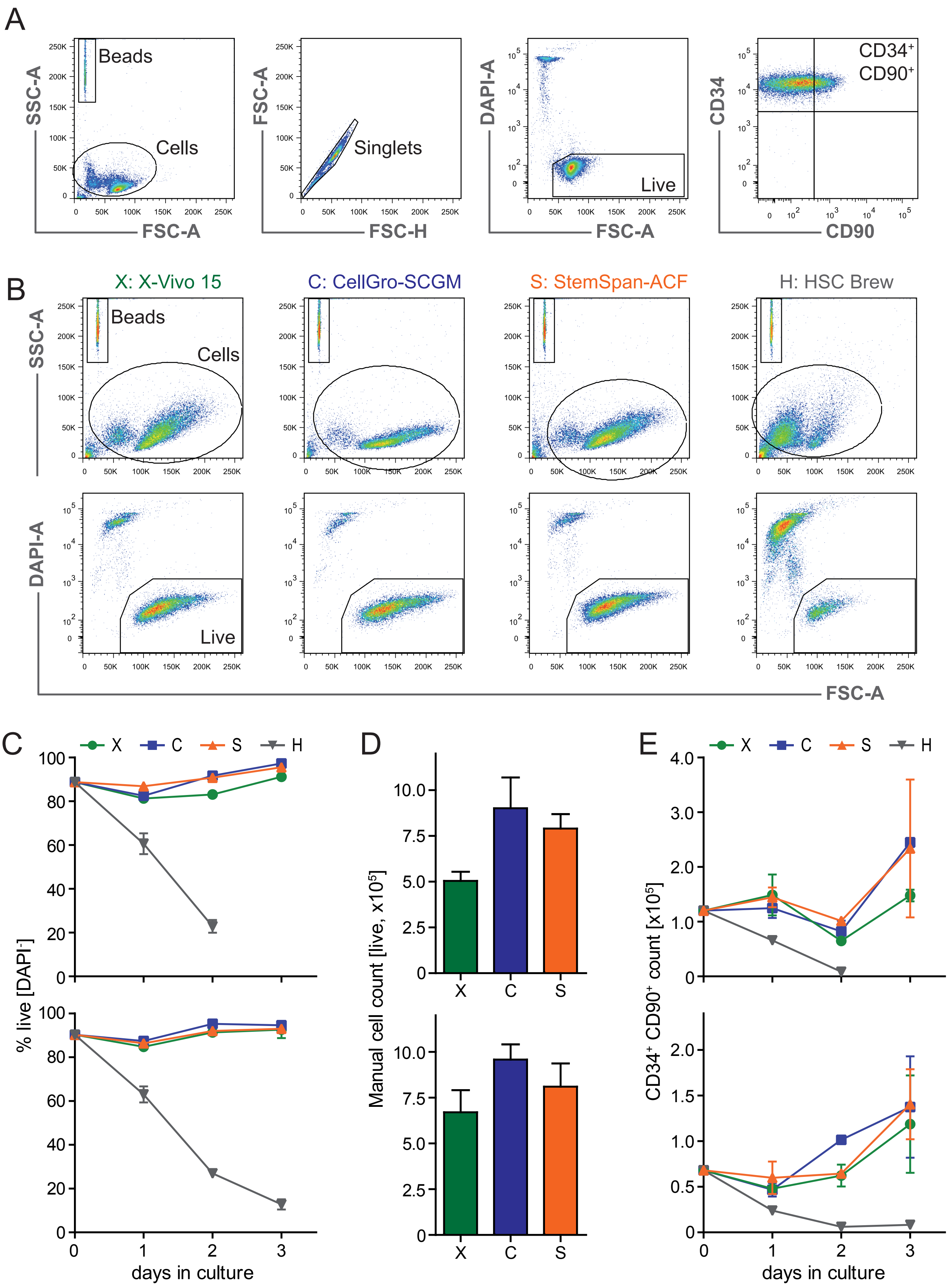
A
Beads
Cells
SSC-A
FSC-A
Singlets
FSC-A
FSC-H
DAPI-A
Live
FSC-A
CD34⁺
CD90⁺
CD34
CD90
B
X: X-Vivo 15
C: CellGro-SCGM
S: StemSpan-ACF
H: HSC Brew
Beads
Cells
SSC-A
DAPI-A
Live
FSC-A
C
X C S H
% live [DAPI⁻]
days in culture
D
Manual cell count [live, x10⁵]
X C S
E
X C S H
CD34⁺ CD90⁺ count [x10⁵]
days in culture

Figure S1. Media comparison for $CD34^+$ HSPC culture and expansion

(**A**) Gating strategy to determine the percentage of the $CD34^+CD90^+$ $HSPC^{prim}$ subpopulation and to determine cell counts using counting beads, exemplarily shown for one of the replicates of donor A on d0. (**B**) Plots showing FCS / SSC profiles of $CD34^+$ HSPCs cultured in the depicted media, and gating strategy to discriminate live cells, exemplarily shown for one of the replicates of donor A on d2. (**C**) Percentage of live cells ($DAPI^-$), as identified by the gating strategy shown in (B), at the indicated time points of culture in the different media. Top panel, donor A; bottom panel, donor B. Error bars: mean of 2 technical duplicates ± SD. (**D**) Manual counts of live cells, determined by trypan blue exclusion on day 3 of culture in the different media. Top panel, donor A; bottom panel, donor B. Error bars: mean of 2 technical duplicates ± SD. (**E**) Counts of $CD34^+CD90^+$ $HSPCs^{prim}$, as identified by FCM using counting beads, at the indicated time points of culture in the different media. Top panel, donor A; bottom panel, donor B. Error bars: mean of 2 technical duplicates ± SD.

A

Transduction enhancer	Site of action	Supplier	Grade	Solvent	Concentration
LentiBOOST	Attachment	SIRION Biotech	Pharma	*pre-dissolved*	1 mg/mL
Prostaglandin E2	Intracellular / post-entry	Cayman Chemical	Pharma	DMSO	10 µM
Protamine sulfate	Attachment	Thermo Fisher Scientific	Pharma	*pre-dissolved*	4 µg/mL
Vectofusin-1	Attachment / fusion	Miltenyi Biotec / Genethon	Pharma	sterile water	10 µg/mL
ViraDuctin	Attachment	Cambridge Bioscience	Research	*pre-dissolved*	1x
RetroNectin	Attachment	TaKaRa Bio	cGMP	*pre-dissolved*	20 µg/mL
Staurosporine^{low}	Intracellular / entry	Cambridge Bioscience	Research	DMSO	400 nM
Staurosporine^{high}	Intracellular / entry	Cambridge Bioscience	Research	DMSO	800 nM
OH-Staurosporine^{low}	*first test as a TE*	MERCK Chemicals	Research	DMSO	400 nM
OH-Staurosporine^{high}	*first test as a TE*	MERCK Chemicals	Research	DMSO	800 nM

B

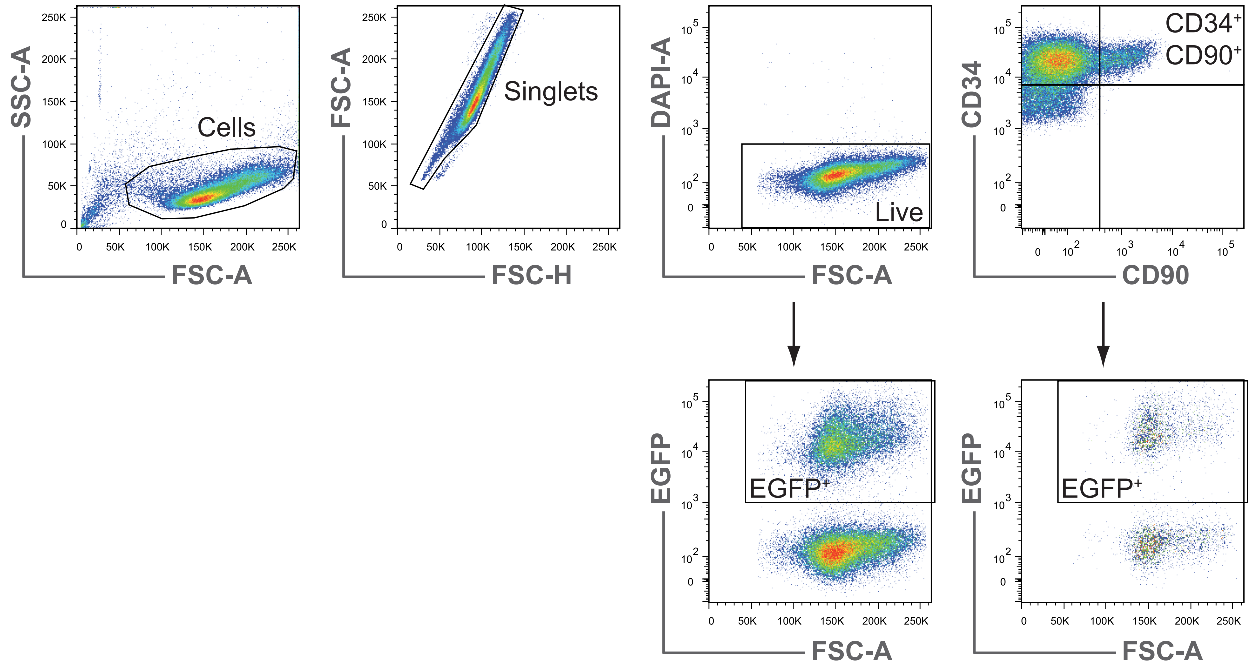

Figure S2. Overview of transduction enhancers and gating strategy
(**A**) Summary of tested transduction enhancers (TEs) and their working concentrations. (**B**) Gating strategy to identify the percentage of live cells, of CD34^{+}CD90^{+} HSPCs^{prim}, and of EGFP^{+} cells within live cell and CD34^{+}CD90^{+} HSPC^{prim} fractions.

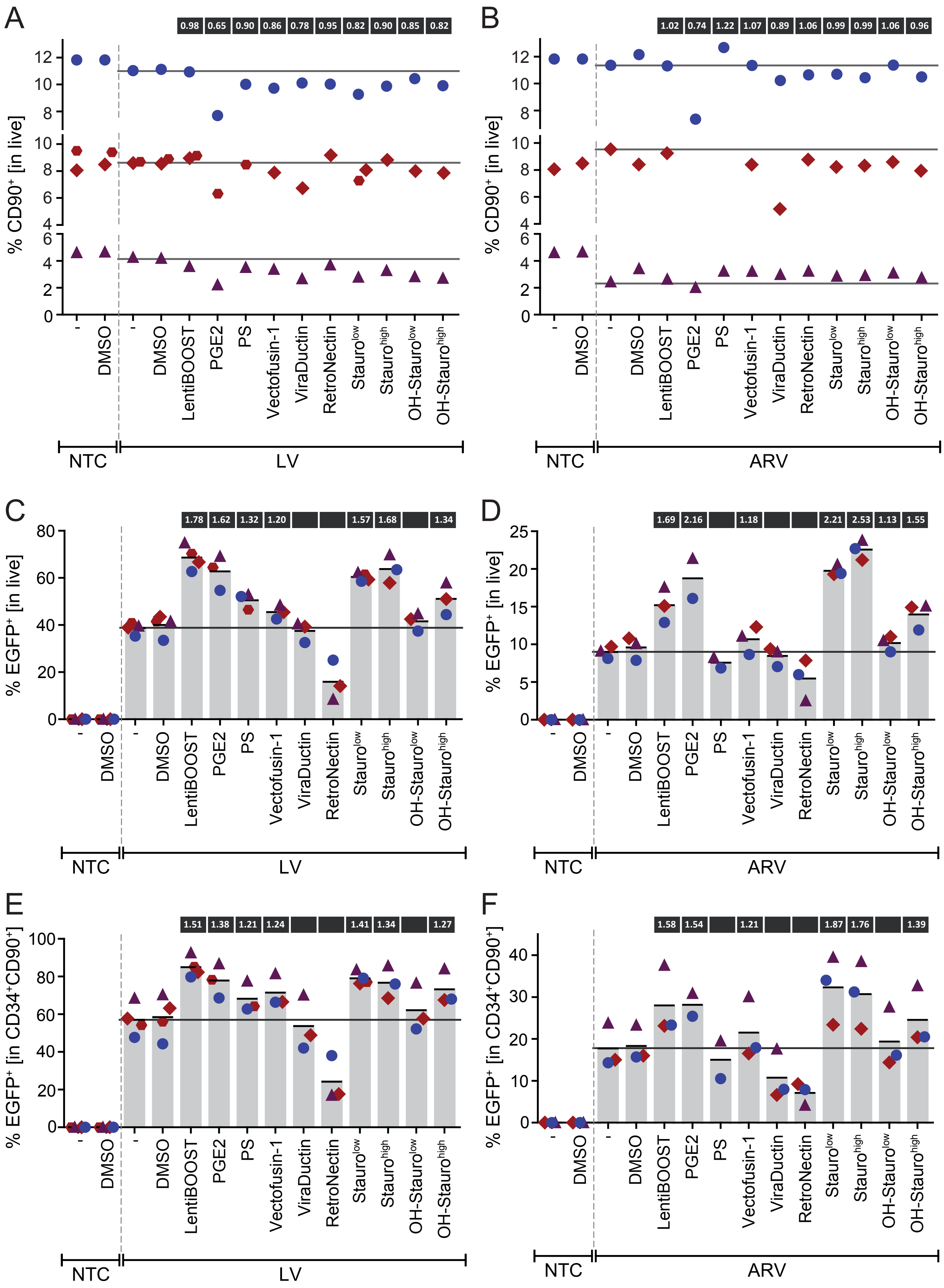
A
B
C
D
E
F
% CD90⁺ [in live]
% EGFP⁺ [in live]
% EGFP⁺ [in CD34⁺CD90⁺]
0.98 0.65 0.90 0.86 0.78 0.95 0.82 0.90 0.85 0.82
1.02 0.74 1.22 1.07 0.89 1.06 0.99 0.99 1.06 0.96
1.78 1.62 1.32 1.20 1.57 1.68 1.34
1.69 2.16 1.18 2.21 2.53 1.13 1.55
1.51 1.38 1.21 1.24 1.41 1.34 1.27
1.58 1.54 1.21 1.87 1.76 1.39
- DMSO - DMSO LentiBOOST PGE2 PS Vectofusin-1 ViraDuctin RetroNectin Stauro^low Stauro^high OH-Stauro^low OH-Stauro^high
NTC
LV
ARV

Figure S3. Single transduction enhancers increase the percentage of CD34$^+$ HSPC transduction with lentiviral and alpharetroviral vectors

(**A**, **B**) Percentage of the CD90$^+$ subpopulation within live cells one week post-transduction of HSPCs with lentiviral (A) and alpharetroviral (B) vectors at MOI 20. NTC, non-transduced control; -, no TE or vehicle; DMSO, vehicle only; PGE2, prostaglandin E2; PS, protamine sulfate; Stauro, staurosporine; OH-Stauro, 7-hydroxy-staurosporine. Donor C was used in 2 independent experiments for selected conditions. Horizontal lines indicate baseline levels in the absence of TE treatment ("-" condition) for each donor. (**C**, **D**) Transduction efficiency, given as the percentage of EGFP$^+$ cells, within live cells one week post-transduction of HSPCs with lentiviral (C) and alpharetroviral (D) vectors at MOI 20. Vertical bars represent the mean from four experiments. Numbers in boxes on top of bars indicate the mean fold increase (if ≥1.1) from the four experiments. (**E**, **F**) Transduction efficiency, given as the percentage of EGFP$^+$ cells, within the CD34$^+$CD90$^+$ HSPC^{prim} fraction one week post-transduction of HSPCs with lentiviral (E) and alpharetroviral (F) vectors at MOI 20. Vertical bars represent the mean from four experiments. Numbers on top of bars indicate the mean fold increase (if ≥ 1.1) from the four experiments.

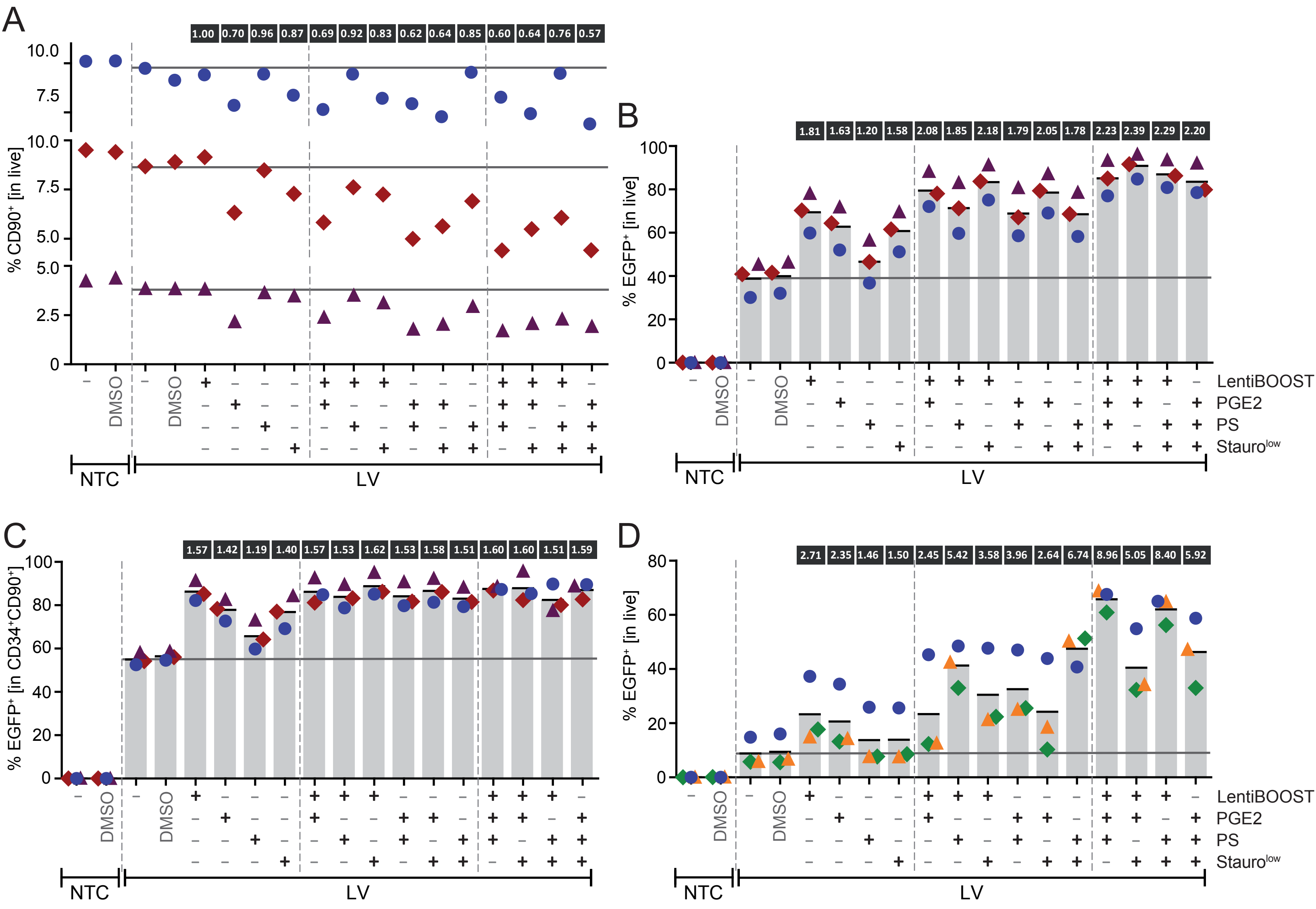

Figure S4. Combination of transduction enhancers increases lentiviral CD34⁺ HSPC transduction over their single use (**A**) Percentage of the CD90⁺ subpopulation within live cells one week post-lentiviral-transduction at MOI 20. NTC, non-transduced control; -, no TE or vehicle; DMSO, vehicle-only control; PGE2, prostaglandin E2; PS, protamine sulfate; Stauro, staurosporine. Horizontal lines indicate baseline levels in the absence of TE treatment ("-" condition) for each donor. (**B**, **C**) Transduction efficiency, given as the percentage of EGFP⁺ cells, within live cells (B) and within the CD34⁺CD90⁺ HSPC^{prim} fraction (C) one week post-transduction with a lentiviral vector at MOI 20. Vertical bars represent the mean from three experiments. Numbers in boxes on top of bars indicate the mean fold increase (if ≥1.1) from the three experiments. (**D**) Transduction efficiency, given as the percentage of EGFP⁺ cells, within live cells one week post-transduction with a lentiviral vector at MOI 10. Vertical bars represent the mean from three experiments. Numbers in boxes on top of bars indicate the mean fold increase (if ≥ 1.1) from the three experiments. Note: for 2 (green and orange symbols) of the 3 donors, staurosporine treatment was performed for 24 h, including the transduction period, instead of the 2-h pre-incubation period chosen in previous experiments.

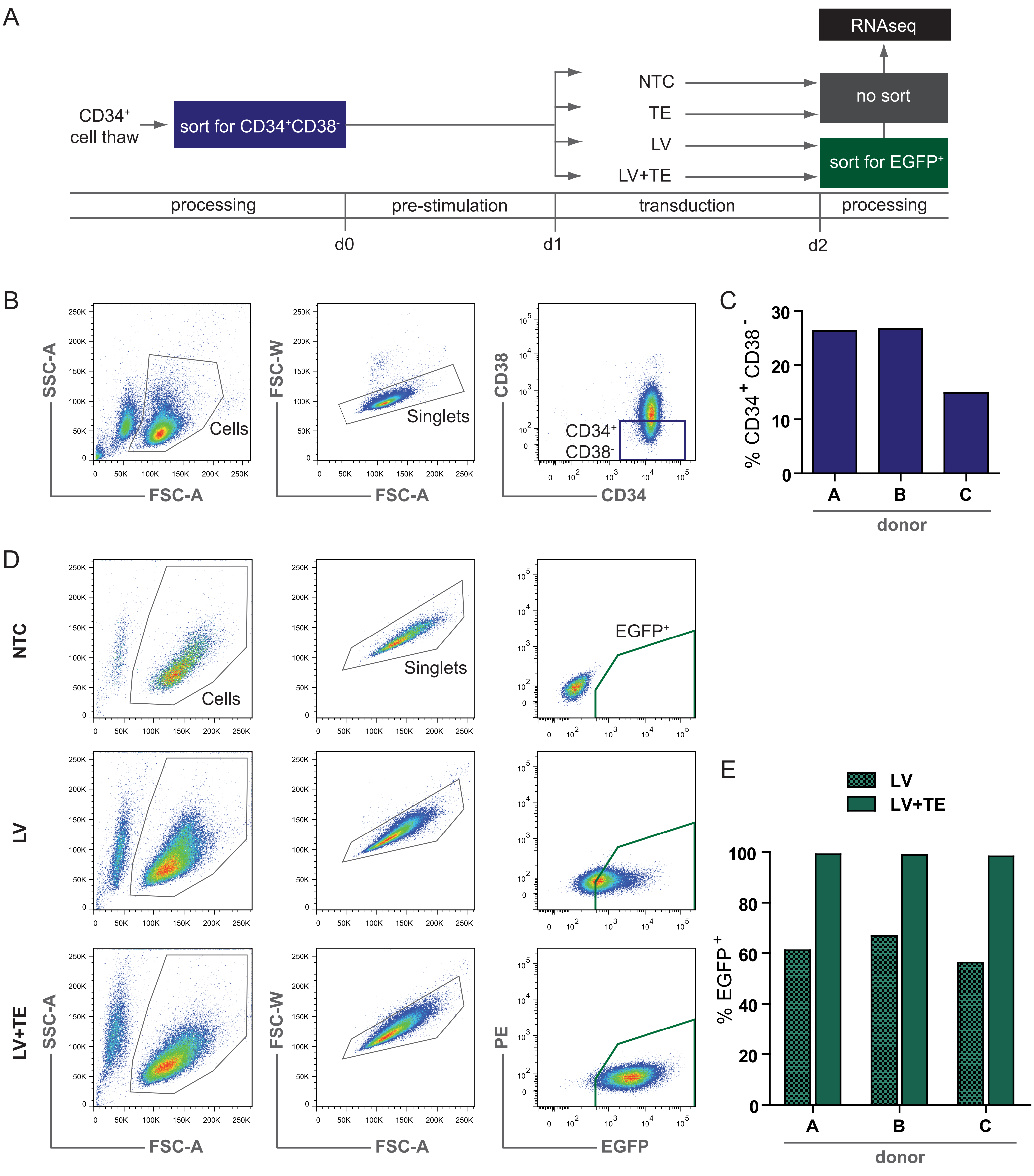

A
CD34+ cell thaw
sort for CD34+CD38-
NTC
TE
LV
LV+TE
no sort
sort for EGFP+
RNAseq
processing
pre-stimulation
transduction
processing
d0
d1
d2
B
SSC-A
FSC-A
Cells
FSC-W
FSC-A
Singlets
CD38
CD34
CD34+ CD38-
C
% CD34+ CD38-
A
B
C
donor
D
NTC
LV
LV+TE
SSC-A
FSC-A
Cells
FSC-W
FSC-A
Singlets
PE
EGFP
EGFP+
E
LV
LV+TE
% EGFP+
A
B
C
donor

Figure S5. Cell sort for RNA-seq

(**A**) Experimental scheme. Purified $CD34^{+}$ HSPCs from 3 healthy donors (A, B, C) were thawed and sorted for $CD34^{+}CD38^{-}$ cells to enrich for more primitive HSPC. The following day, transduction using the lentiviral EGFP-encoding vector as shown in Figure 2A was performed at MOI 20 in the presence (LV + TE) or absence (LV) of the transduction enhancers LentiBOOST plus PS. Non-TE-treated, non-transduced cells (NTC) and TE-treated, non-transduced cells (TE) served as controls. The day after transduction, transduced cells were sorted for the $EGFP^{+}$ cell population and aliquots were frozen for RNA extraction and RNA-seq. Non-transduced controls were frozen unsorted. (**B**) Gating to identify $CD34^{+}CD38^{-}$ cells in the cell sort on d0. (**C**) Percentage of $CD34^{+}CD38^{-}$ cells in the cell sort on d0. (**D**) Gating to identify $EGFP^{+}$ cells in the cell sort on d2, exemplarily shown for one donor. (**E**) Percentage of $EGFP^{+}$ cells in the cell sort on d2. LV, transduced in the absence of transduction enhancers; LV + TE, transduced in the presence of LentiBOOST plus PS.

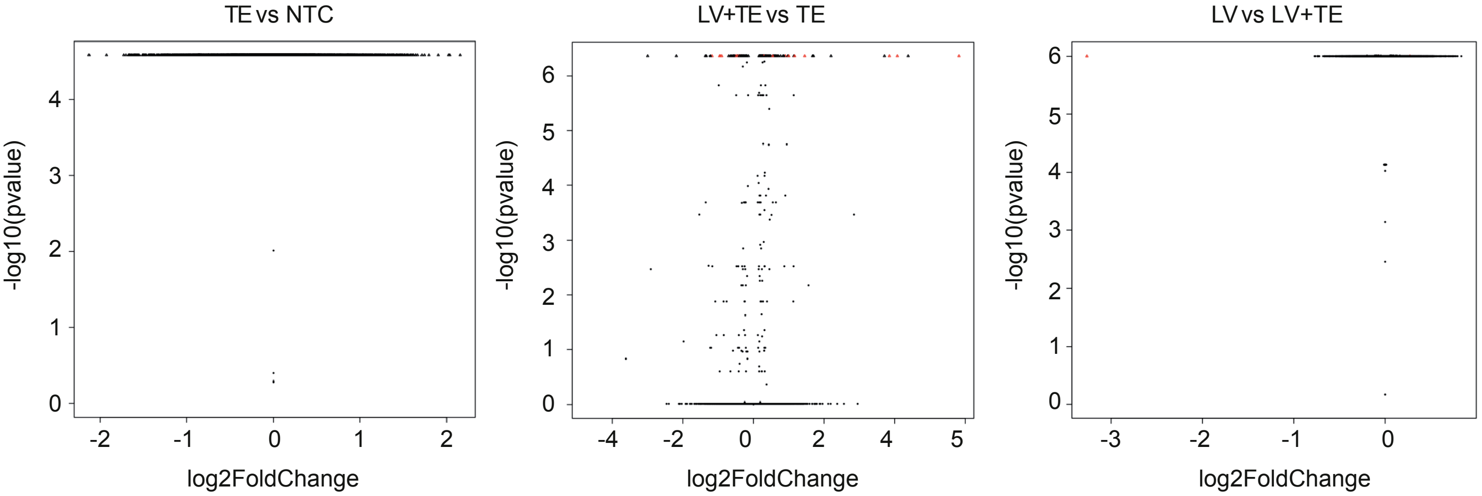

Figure S6. RNA-seq analysis

Volcano plots of RNA-seq data from three biological replicates; differentially expressed transcripts (adjusted p-value ≤ 0.05) between the different conditions are highlighted in red.

SUPPLEMENTAL MATERIALS AND METHODS

Cell culture

Human embryonic kidney (HEK293T) cells were cultured in Dulbecco's Modified Eagle's Medium (DMEM) + GlutaMAX^{TM}-I supplemented with 10% heat-inactivated fetal bovine serum (FBS) and 1 U/mL penicillin plus 100 µg/mL streptomycin (all Gibco^{TM}). Human colorectal adenocarcinoma HT-29 cells were kept in Roswell Park Memorial Institute (RPMI)-1640 + GlutaMAX^{TM}-I supplemented with 10% heat-inactivated FBS and 1 U/mL penicillin plus 100 µg/mL streptomycin (all Gibco^{TM}).

Vector design

A lentiviral (LV) SIN EGFP-reporter vector, pRRLSIN.cPPT.PGK-GFP.WPRE, was purchased from Addgene (Cat. #12252). To facilitate cloning of the alpharetroviral (ARV) SIN EGFP-reporter vector pAlpha.SIN(noTATA).hPGK.EGFP.WPRE, an entry vector, pAlpha.SIN(noTATA), was cloned. For this, a gene string (Invitrogen) introducing a new multiple cloning site (MCS) directly 5' to the PRE, and encompassing the complete PRE sequence plus the 3' SIN LTR, was cloned into pCR-Blunt II-TOPO (Invitrogen). The gene string additionally introduced a point mutation to wipe out the HindIII restriction site flanking the PRE at its 3' end. Sequence identity of the gene string was verified by sequencing. The entire cassette was then excised using HpaI and XhoI restricition sites chosen to flank the gene string's ends and introduced into pAlpha.SIN(noTATA).EFS.EGFP.WPRE,[1] which was cut with ClaI, blunted, and then cut with XhoI, to create pAlpha.SIN(noTATA).Entry.WPRE. A hPGK-EGFP cassette was excised from pRRLSIN.cPPT.PGK-GFP.WPRE and introduced into pAlpha.SIN(noTATA).Entry.WPRE using EcoRV and SalI restriction sites to create pAlpha.SIN(noTATA).hPGK.EGFP.WPRE.

Vector production

For LV vector particle production, standard transient PEI-mediated co-transfection of HEK293T cells with the transfer vector plasmid and packaging components (PlasmidFactory, Bielefeld, Germany) was employed.[2] In brief, a total of 12 x 10^6 cells were plated per T175 flask the day before transfection. For transfection, the following DNA amounts were added to 5 mL of Opti-MEM I Reduced Serum medium (Gibco^{TM}) and filtered: vector construct (40 µg), pMD.G (10 µg, encoding for the VSV-G pseudotype), and pCMV.ΔR8.74 (30 µg, encoding for LV Gag-Pol). To 5 mL of Opti-MEM I, 1 µl of a 10 mM stock of PEI was added and filtered. DNA and PEI solutions were mixed 1:1 and incubated for 20 min at room temperature. Cells were washed in Opti-MEM I, and 10 mL of the DNA-PEI mix were added per flask. Medium was changed to DMEM-based complete culture medium after 4 h. Supernatant was harvested 48 h and 72 h post-transfection, pooled, filtered through a 0.22 µm pore size filter, and concentrated via ultracentrifugation for 2 h at 23,000 rpm (rotor SW32Ti) and 4°C. Pellets were resuspended in basic SCGM without supplements and stored at -80°C until usage.
ARV vector particles were produced by standard calcium-phosphate-based transient co-transfection of HEK293T cells with the transfer vector plasmid and packaging components as previously described.[3] Briefly, 5 x 10^6 cells were plated per 10 cm dish the day before transfection. Using the CAPHOS Calcium Phosphate Transfection Kit (Sigma), the following DNA amounts were transfected in the presence of 25 µM chloroquine (Sigma): 5 µg of vector construct, 1.5 µg of pMD.G, and 2.5 µg of pcDNA3.Alpha.gag/pol.CO (encoding for codon-optimized ARV Gag-Pol). During transfection and particle production, the medium was supplemented with 1.5% HEPES (Gibco^{TM}). Supernatants were collected 36 h and 48 h post-transfection, filtered through a 0.45 µm pore size filter, and concentrated via ultracentrifugation for 2 h at 23,000 rpm (rotor SW32Ti) and 4°C. Pellets were resuspended in basic SCGM and stored at -80°C until further usage.

Titers were determined in triplicate by transduction of HT-29 cells with serial vector dilutions. Cells were harvested 3-6 days post-transduction and mean vector copy numbers per cell determined by quantitative real-time PCR. Titers

(infectious genomes (IG) per mL) were calculated based on the volume of vector applied, the cell count on the day of transduction, and the mean vector copy number.

The SCID-X1 vector CCL_pEF1a_IL2RGcoWPRE* manufactured to cGMP was produced by Yposkesi (Corbeil-Essonnes, France) by transient transfection of HEK293T cells. The culture media was harvested after transfection and treated in different ways to ensure any impurities are removed. Finally, the harvest was concentrated and formulated in X-Vivo 20, filtrated and aseptically filled into 1mL vials.

Vector copy number (VCN) determination

Cells were harvested on day 14 post-transduction. Genomic DNA was extracted using the DNeasy Blood and Tissue Kit (Qiagen) as per manufacturer's instruction. VCNs were determined by TaqMan-based quantitative real-time PCR using primers and probes specific to the PRE sequence of the vector (PRE_FW 5'-GAGGAGTTGTGGCCCGTTGT-3', PRE_RV 5'-TGACAGGTGGTGGCAATGCC-3', PRE_probe 5'-FAM-CTGTGTTTGCTGACGCAAC-TAMRA-3')[4] and to the human Albumin (ALB) reference gene (hALB_FW 5'-GCTGCTATCTCTTGTGGGCTGT-3', hALB_RV 5'-ACTCATGGGAGCTGCTGGTTC-3', hALB_probe 5'-VIC-CCTGTCATGCCCACACAAATCTCTCC-TAMRA-3'). PCR reactions were carried out on a CFX96 Touch Real-Time System (Bio-Rad, Watford, UK) with ABsolute QPCR ROX Mix (Thermo Fisher Scientific) and the following PCR program: 15 min at 95°C, followed by 40 cycles of 15 sec at 95°C / 30 sec at 55.6°C / 1 min at 72°C. A standard plasmid carrying the PRE and human Albumin target sequences served for absolute quantification of the mean VCN per cell.

For the GMP experiment, a QC vial was frozen together with the cell product. This vial was thawed and a liquid culture was started. Cell pellets were harvested on day 7 following the SOP for this particular clinical trial. Genomic DNA was extracted using the DNeasy Blood and Tissue Kit as per manufacturer's instruction. VCNs were determined by quantitative real-time PCR using primers and probes specific to the LV PSI sequence (PSI_FW 5'-CAGGACTCGGCTTGCTGAAG-3'; PSI_RV 5'-TCCCCCGCTTAATACTGACG-3'; PSI_probe 5'-FAM-CGCACGGCAAGAGGCGAGG-TAMRA-3') and to the human albumin reference gene (sequences as above). All reactions were performed with ABsolute qPCR ROX Mix and run in the CFX96 Touch Real-Time PCR Detection System under the following conditions: 10 min at 95°C and then 40 cycles of 15 sec at 95°C and 1 min at 60°C. The reference standard was obtained by serially diluting a plasmid carrying a single copy of the PSI sequence and the albumin gene.

For CFU, VCNs were determined by TaqMan-based quantitative real-time PCR for the simultaneous detection of LV PSI using primer and probe sequences as stated above versus the human cellular albumin (ALB) gene (ALB_FW 5'-GCTGTCATCTCTTGTGGGCTGT -3'; ALB_RV 5'-ACTCATGGGAGCTGCTGGTTC-3'; ALB_probe 5'-CCTGTCATGCCCACACAAATCTCTCC-3'). Reactions used the PerfeCTa® FastMix® II Low ROX (Quantabio, Beverly, MA) and the 7500 Real-Time PCR System running SDS v2.4 and the standard PCR program of 10 min at 95 °C, followed by 40 cycles of 15 sec at 95°C, 1 min at 60°C (Applied Biosystems). The PCR was calibrated by a standard curve made by serial dilutions of a plasmid carrying a single copy of the vector bearing HIV and of the ALB sequences. The assay also included gDNA from clones with known copy number as controls.

Colony forming unit (CFU) assay

Cells were plated in MethoCult™ H4435 Enriched medium (StemCell Technologies) as per manufacturer's instruction, using a density of 125 cells in a total volume of 1.1 mL per well of a 6-well plate. CFUs were enumerated by manual counting on day 14 post-plating, discriminating BFU-E, CFU-GM and CFU-GEMM.

For the GMP experiment, a QC vial was frozen together with the cell product. This vial was thawed and a CFU assay was set up as described above. Individual colonies were picked and transferred to a 96-well plate containing lysis buffer (Proteinase K (0.91 mg/ml), Tween 20 (0.5%), Nonidet P40 (0.5%)). The plate was heat treated for DNA extraction and the VCN was determined.

SUPPLEMENTAL REFERENCES

1. Moiani, A, Suerth, JD, Gandolfi, F, Rizzi, E, Severgnini, M, De Bellis, G, *et al.* (2014). Genome-wide analysis of alpharetroviral integration in human hematopoietic stem/progenitor cells. *Genes* **5**: 415–429.

2. Santilli, G, Almarza, E, Brendel, C, Choi, U, Beilin, C, Blundell, MP, *et al.* (2011). Biochemical Correction of X-CGD by a Novel Chimeric Promoter Regulating High Levels of Transgene Expression in Myeloid Cells. *Mol. Ther.* **19**: 122–132.

3. Suerth, JD, Maetzig, T, Galla, M, Baum, C and Schambach, A (2010). Self-Inactivating Alpharetroviral Vectors with a Split-Packaging Design. *J. Virol.* **84**: 6626–6635.

4. Rothe, M, Rittelmeyer, I, Iken, M, Rüdrich, U, Schambach, A, Glage, S, *et al.* (2012). Epidermal growth factor improves lentivirus vector gene transfer into primary mouse hepatocytes. *Gene Ther.* **19**: 425–434.
